# Temporal divergence of changes in pain and pain-free grip strength after manual acupuncture or electroacupuncture: an experimental study in people with lateral epicondylalgia

**DOI:** 10.1186/s13020-017-0143-z

**Published:** 2017-08-04

**Authors:** Jaewon Jeon, Erin Bussin, Alex Scott

**Affiliations:** Centre for Hip Health and Mobility, Vancouver Coastal Health Research Institute and Department of Physical Therapy, University of British Columbia, 2635 Laurel Street, Vancouver, BC V5Z3M9 Canada

**Keywords:** Acupuncture, Tendinitis, Tendon, Tendinopathy, Musculoskeletal

## Abstract

**Background:**

The objective of this study was to examine, in individuals with lateral epicondylalgia (LE), the acute time course of acupuncture-induced hypoalgesia and change in pain-free grip strength (PFGS).

**Methods:**

This was an experimental study, conducted at a single research center in Vancouver, BC. Twenty-one participants with unilateral LE lasting more than 6 weeks duration were enrolled. Participants received a single treatment of acupuncture (either electroacupuncture, 10–30 Hz, or manual acupuncture, assigned randomly). The primary outcome measure was pain level (0–10) during tendon loading (while making a fist) immediately after treatment, and over a 72 h follow-up period. Secondary outcome measures included pain-free grip strength (N).

**Results:**

There was a small but statistically significant reduction in participants’ perceived pain level immediately after acupuncture (mean improvement of 1.2, 95% CI 0.45–1.9). This change in pain was not accompanied by a change in PFGS. No difference was observed between the two types of acupuncture at any time point.

**Conclusions:**

The use of acupuncture or electroacupuncture, as administered in the current study, is unlikely to acutely enhance the ability of people with LE to engage in pain-free rehabilitation exercise.

*Trial registration* Registered February 25, 2015. ISRCTN14667535, http://www.isrctn.com/ISRCTN14667535

**Electronic supplementary material:**

The online version of this article (doi:10.1186/s13020-017-0143-z) contains supplementary material, which is available to authorized users.

## Background

Lateral epicondylalgia (LE, or tennis elbow) is a condition characterized by lateral elbow pain and pathology in the common wrist extensor tendons. Pathology is visible on ultrasound as swelling, disorientation of collagen fibre bundles, and increased blood flow [1, 2]. Under the microscope, the pathology is heterogeneous, containing areas of angiofibroblastic reaction as well as acellular regions of degenerate morphology [3]. The pain of LE is thought to involve nociception from the pathological tendon and associated tissues [4, 5], along with peripheral and central sensitization leading to widespread mechanical hyperalgesia [2, 6], with or without referred pain [7]. People with LE experience various types of pain, including resting pain (which may disturb sleep), as well as pain which is worsened by gripping activities such as holding a cup or carrying a bag [8]. A contemporary model of LE emphasizes the interactions among three pathophysiological processes: tissue pathology, changes in the pain system, and changes in motor function [9]. It has been proposed that integrated treatment for people with LE should address observed changes in all three of these domains [9].

The majority of patients with chronic LE recover within 1 year, with or without intervention [10], however resistance exercise has been shown to improve the medium-term outcomes for patients with LE [11]. Multimodal physiotherapy including resistance training has also been found to be superior to a wait-and-see approach in the medium term [10], and is associated with fewer recurrences in the long term than corticosteroid injections [12]. However, pain can hinder active participation in rehabilitation [13]. Exercise programs are, for that reason, often combined with hypoalgesic therapies (e.g. bracing/taping, manual therapy) to allow greater loads to be prescribed without exacerbating pain [14]. Some authors [15] have suggested combining acupuncture with an exercise-based rehabilitation program, however there is no study yet which has examined the potential utility of this approach for people with LE.

Acupuncture has been systematically reviewed with regard to its ability to induce analgesia for a variety of conditions, including fibromyalgia [16], cancer pain [17], peripheral joint OA [18], idiopathic bowel syndrome [19], ankle sprain [20], chronic pain in people with spinal cord injury [21], migraine [22], shoulder pain [23], rheumatoid arthritis [24], low back pain [25], labour pain [26], and lateral epicondylalgia [27]. Of these conditions, sufficient evidence on which to base conclusions was reported for fibromyalgia and chronic episodic migraine. For migraine, acupuncture was more successful than sham needling at reducing the frequency of headaches, although the effect was noted to be small [22].

Acupuncture is one of the treatments that has been suggested to provide short-term analgesia for people with LE [28], although the Cochrane collaboration has concluded that the evidence to support this treatment approach is limited by a high risk of bias [27]. The clinical approach for people with LE depends on the practitioner’s training and approach to assessment (e.g. TCM vs medical acupuncture) and also varies depending on an individuals’ symptoms. Treatment typically involves the insertion of several small gauge needles to a depth of 1 cm or more into points in the vicinity of the painful area, and can also include needling into tender spots of associated musculature, as well as regional points including the neck and bilateral upper and lower extremities [29]. Acupuncture stimulates afferent nerve pathways [30], culminating in the activation of neurons in the brain related to pain processing [31]. Early studies in humans (e.g. [32]) showed a significant role of endogenous opioids in acupuncture-induced analgesia, but this has not been consistently observed by subsequent studies [33, 34]. Electroacupuncture (EA; the stimulation of sensory nerves using electrical current delivered via acupuncture needles) may invoke additional analgesic mechanisms compared to needling alone; animal studies have suggested a relation between the frequency of electrical stimulation (in Hz) and the amount of opioids released in the brain and spinal cord [35]. An additional proposed mechanism of acupuncture, consistent with fMRI studies, includes the inhibition of ascending nociceptive information (discussed in [31]). In spite of the above, there are no studies which have directly examined the impact of EA and manual acupuncture (MA) for LE using clinically relevant outcome measures for pain and motor function, while controlling important sources of bias. A key paper [36] reported an average reduction in pain of 55.8% in 24 LE participants treated with manual acupuncture. Pain relief was maximal immediately after treatment, and displayed an average duration of 20 h. The key finding of this paper (immediate pain relief for LE in 79% of the patients treated with non-segmental acupuncture, GB34, 5 min treatment duration, peaking immediately after treatment) has never been replicated to our knowledge. Furthermore, the impact of acupuncture on elbow function has not been examined.

PFGS is a sensory threshold test for mechanically-induced pain in people with LE, with excellent reliability, validity, and sensitivity to change [37]. PFGS provides the clinician a method to monitor the threshold of pain onset during gripping (in N) during rehabilitation [37]. Despite this, a recent systematic review of the effects of acupuncture on sensory thresholds did not identify any studies which used PFGS as the outcome measure [38]. In particular, no study to our knowledge has examined acute changes in PFGS following acupuncture treatment of LE, as exists for bracing [39], taping [40], or manual therapy [41] (all of which induce clinically meaningful levels of hypoalgesia in people with LE). It would be useful to know whether acupuncture has a similar magnitude and time course of effect as these other modalities, as this knowledge would inform current models of clinical reasoning for rehabilitation of people with LE [15].

The overall objective of this study was to explore the acute time course of improvements in pain and PFGS in people with LE following a treatment of MA or EA. We hypothesized that both MA and EA would induce a small, short-term hypoalgesic effect, and that this effect would be associated with a simultaneous improvement in PFGS.

## Methods

### Study design

The Minimum Standards of Reporting Checklist (Additional file 1) contains details of the experimental design, and statistics, and resources used in this study.

This was a randomized, double-blinded, parallel-group experiment conducted at the Centre for Hip Health and Mobility in Vancouver, British Columbia, Canada. This study started in May 2015 and ended in December 2015. All outcome measures which were collected are reported here.

### Recruitment and eligibility criteria

Participants were recruited from the general community by advertising in community centres, gyms, tennis facilities, medical clinics and coffee shops in Vancouver, British Columbia. Interested participants contacted the study team either by telephone or email and underwent a telephone interview to screen for the minimal eligibility criteria. A consent form was emailed to those who met the eligibility criteria; and individuals who decided to participate were invited to the Centre for Hip Health and Mobility for further screening and treatment.

Inclusion criteria were: (1) unilateral elbow pain at or around the lateral epicondyle of the humerus for more than 6 weeks, (2) elbow pain provoked by palpation of the lateral humeral epicondyle and by gripping, (3) elbow pain triggered by either resisted wrist extension or resisted middle finger extension [15], between 19 and 65 years old, and (4) fluent in English.

Exclusion criteria were: (1) any other concurrent neck, shoulder, elbow, or arm disorders, (2) history of any injections for LE (e.g. corticosteroid, autologous whole blood, PRP, or prolotherapy), (3) received acupuncture for LE, (4) pregnant, (5) pacemakers or other electrical device implanted in the body, (6) history of seizures or epilepsy, (7) untreated hemorrhagic disorders, (8) infected tissues, osteomyelitis, or wounds around the elbow, (9) active deep vein thrombosis or thrombophlebitis, (10) impaired sensation at and around the elbow, (11) not able to give accurate and timely feedback due to cognition or communication impairment, (12) cancerous lesions at or around the elbow, (13) radiation therapy around the elbow within the previous 6 months, (14) impaired circulation around the elbow, (15) surgery or bone fracture at or around the elbow previously, (16) needle phobia, (17) inflammatory rheumatic diseases, and (18) opioid medications.

### Sample size

For this pilot study, we actively recruited over an 8-month period. 21 adults with LE participated. All available participants during this period that met all the eligibility criteria were included in this study. To detect a reduction in pain during gripping of 30% (which is a provisional benchmark for a moderately important clinical change [51]) with a SD of 40 and power of 0.80, a sample size of 16 was required. This calculation was based on a within-group change over time.

### Procedures and interventions

After obtaining written informed consent from participants, they were screened for their eligibility for the study by a clinician (JJ).

Before their first visit, participants were asked to: (1) refrain from taking NSAIDs or analgesia for 3 days prior to the study until its completion; (2) avoid any therapy for 3 days prior to the study until its completion; (3) avoid excessive caffeine intake (no more than 2 cups of coffee) 24 h before the study and during the 72 h follow-up; and (4) avoid any activities that aggravate elbow pain for 72 h after treatment.

After the baseline measurements, participants received acupuncture on LI 4, TE 5, LU 5, LI 10, LI 12, SP 6, and GB 34 [36, 42–44]. Needling was done only on the affected arm and ipsilateral leg. Only sterile, single-use, disposable stainless steel needles were used (Seirin L-type, 40 mm × 0.25 mm) for both treatment groups. Participants’ skin at the needling sites was cleaned with antiseptic prior to needling. After inserting acupuncture needles into the skin to the required depth (1.5–3.5 cm), the clinician manipulated the acupuncture needles until the patients reported deqi (“arrival of qi”). Deqi is a subjective feeling of a patient at the region where an acupuncture needle is inserted into the acupoint and it is characterized by aching, soreness, numbness, tingling, pressure, or heaviness—this is typically ascribed to stimulation of peripheral sensory nerves [45].

Participants who were allocated in the EA group received electro-stimulation between two pairs of acupoints: one pair between LI 10 (−ve lead) and LI 12 (+ve lead) and the other pair between LI 4 (−ve lead) and SJ 5 (+ve lead). Electro-stimulation was applied using an electro-acupuncture unit (ES-160) (ITO physiotherapy and rehabilitation, Japan). The frequency was set as alternating between 10 and 30 Hz (alternating between two frequencies every 3 s). The pulse width was set as 400 μs. The intensity was set and maintained at a point at which participants received a sensory stimulus, such as tingling or numbness sensation, without visible muscle contraction. Electro-stimulation was applied for 30 min [46, 47].

Participants in the MA group received sham EA: the wires with metal clips were connected to acupuncture needles the same way as in EA group. However, the intensity dials that were connected to these two wires were not manipulated. Instead, the other intensity dials, which were not connected with wires, were turned on so that the ES-160 would still make stimulus sounds as if participants were receiving electrical stimulation. Participants were told that they may or may not feel a tingling sensation due to the use of micro-currents in the study. The electro-stimulator was placed in an opaque box so that participants were not able to watch how the clinician controlled the machine. In order to find out whether the blinding method was successful or not, participants were asked in the survey “Which group do you think you were belonged to, the EA or MA group?” at the end of the their participation.

Acupuncture needles were left in place for 30 min in both treatment groups. Acupuncture points located on the leg (GB 34, SP 6) were re-stimulated with a twirling method, in both treatment groups, once during the middle of treatment. Manipulation of the needles during the treatment was to stimulate acupuncture points, which in turn stimulates peripheral sensory nerves to create pain relief. All acupuncture was performed by a clinician (JJ) who was a registered acupuncturist in British Columbia with over 10 years of experience in clinical practice and teaching acupuncture.

A randomization list of 20 equally weighted, unblocked allocations was uploaded to clinical data management software (REDCAP) prior to the start of the study. The randomization sequence was concealed from the clinician and assessor, and the randomization was stratified by sex. To avoid clinician bias, the participants’ treatment group allocation was assigned after all the treatment steps were completed, including insertion of the acupuncture needles, with the exception of the final step of applying the electro-stimulation current. After placement of the acupuncture needles by the clinician (JJ), a third person who was not involved in this study was called to the treatment room to witness the completion of needling and the randomization process by pressing a button on a computer using the REDCAP software.

### Baseline and outcome measurements

Prior to receiving treatment, participants’ demographic information, the level of pain and functional disability (PRTEE questionnaire), and the level of pain-related fear of movement (TSK-11 questionnaire), were recorded independently by the participants.

PFGS of unaffected and affected arms with LE were taken by a third, blinded investigator (EB) at baseline, immediately after treatment, and 24 and 72 h after the treatment. For PGFS (and the other outcome measures), 72 h was selected based on the expected duration of analgesia as observed by Molsberger and Hille [36]. Each measurement was repeated three times at 30-s intervals starting with the unaffected arm. Effort to get three consistent readings with less than 10% discrepancy between measurements was made by measuring PFGS more than three times if needed. To avoid bias, encouragement was not given at any time during PFGS measurements and participants were blinded to their PFGS readings. PFGS was measured with an electronic digital grip dynamometer (MIE Medical research, UK). Participants were in the supine position with the arm at the side (slight abduction), elbow extended, forearm pronated, and wrist extended. When testing PFGS, we ensured that the dynamometer and forearm were fully supported on the table top. For measurements in the affected arm, participants were instructed to apply force gradually on the grip and stop squeezing as soon as pain was felt; if participants had pain at rest, they were asked to squeeze until the point where their pain first increased; and for measurement of the unaffected arm, they were asked to squeeze the grip maximally.

Perceived pain level was recorded on an 11-point numeric rating scale (NRS) by a third investigator (EB). Participants were instructed to squeeze their fist as tightly as possible, and rate their resulting pain from 0 to 10, with 10 being the worst imaginable pain. This method of rating pain during a provocative maneuver was selected because (a) many people with LE do not have any resting pain, (b) the method avoids any manipulation or palpation of the elbow by an assessor, and (c) the method avoids any recall bias associated with rating average or worst pain during the previous week. The NRS has shown to a reliable tool for measuring pain in patients with musculoskeletal disorders of upper limb [48].

Acupuncture treatment, measurement of PFGS, and numeric rating of pain were all conducted in the same (supine) position. Before and after acupuncture treatment, PFGS was evaluated followed by pain rating.

Participants were provided with pain diaries to fill out after leaving the research centre, and asked to rate their pain 3 times a day (morning immediately on waking, midday, bedtime) for 72 h following treatment. For self-measurement, participants were instructed to stand straight and position their arms hanging at the side of their body, with their elbow extended, forearm pronated, and wrist extended, make a fist as tightly as possible, then rate their pain. Verbal instructions were given with demonstrations to participants onsite, and the written and visual instructions were listed on the first page of their pain diary.

The GROC scale is a reliable and valid measure of patient’s perceived level of change in their condition over a period of time, commonly used in clinical research and practice [49]. Although we considered it unlikely that there would be any significant clinical improvement over the brief course of our experiments, at the 24 h time point participants were asked “Over the past 24 h since you received acupuncture treatment, how has your condition changed with respect to your elbow pain?” and were asked to rate their condition on a seven-point Likert type scale—much better, moderately better, slightly better, unchanged, slightly worse, moderately worse, and much worse.

At the conclusion of the study, participants were asked to guess their group allocation and report their confidence level with the answer. They had choices of ‘Not confident at all’, ‘Somewhat confident’, ‘Neutral’, ‘Confident’ and ‘Very confident’. In addition, they were questioned: (1) whether they needed to take NSAIDS or any other pain killers for pain control; (2) whether they avoided receiving any therapy; and (3) whether they avoided any activities that aggravate their elbow pain during their participation period.

### Statistical analysis

Linear mixed-effects models (repeat measures ANOVA) were used to determine whether there were changes in pain-free grip strength and perceived pain level over a 72-h period after one treatment of either EA or MA, and whether this change differed between the two treatment groups. Pain levels measured on site and at home (via diaries) were considered as separate outcomes (due to the variation in testing position necessitated by the experimental design), and were therefore analyzed in separate models. The independent variables were time (within-participants) and treatment (between-participants). Although this was not the primary goal of the study, the impact of potential covariates (hand dominance, age, sex, duration of LE, TSK score, PRTEE total score and subscores) were included in the statistical model to determine their potential influence on the change in pain or change in PFGS. P values were adjusted using the Bonferroni method, and P values of less than 0.05 were considered statistically significant. Analysis was performed by an independent statistician at UBC.

## Results

### Recruitment, allocation, and drop-outs

**Fig. 1:**
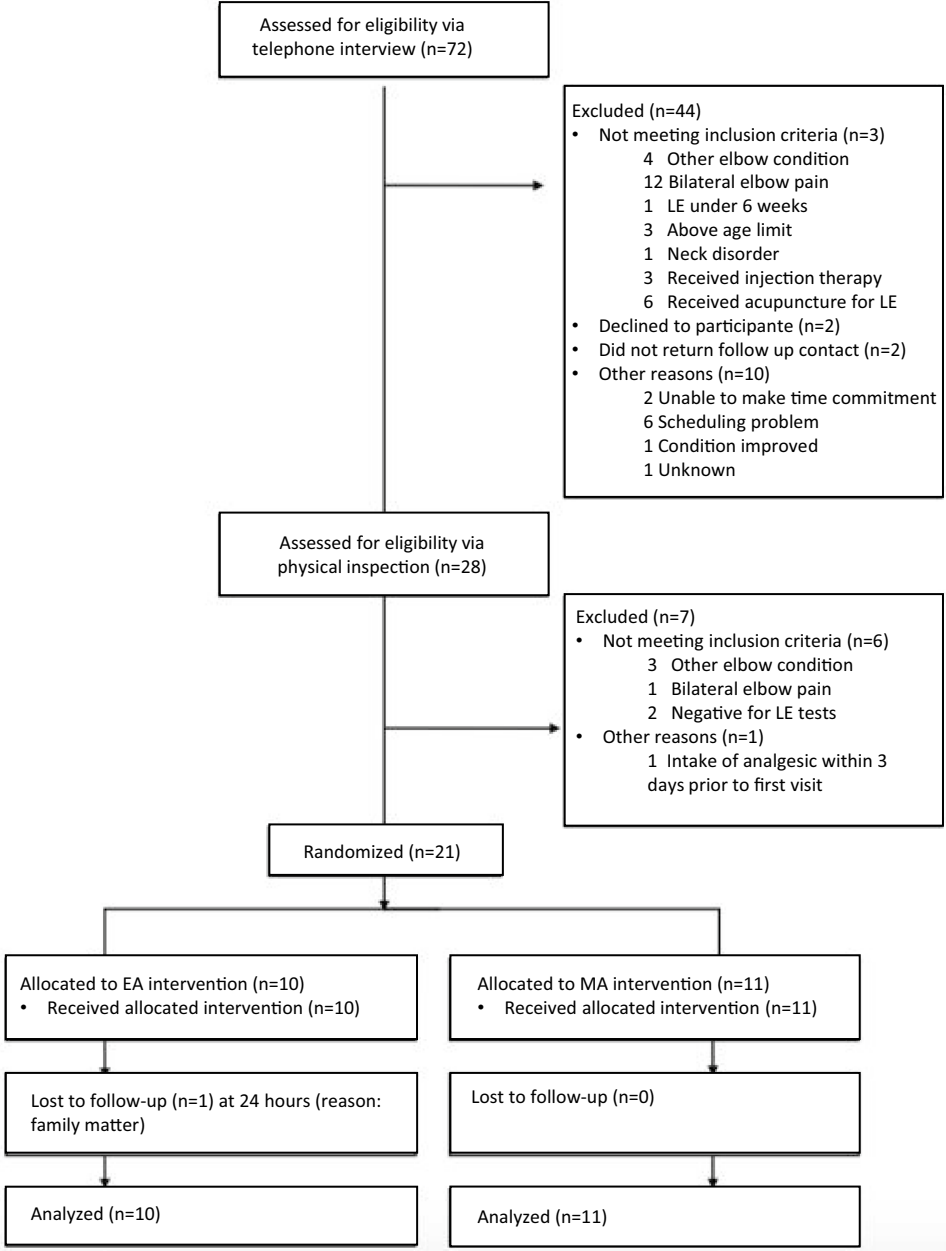
Flow of participants

During the period May to December 2015, twenty-one participants were enrolled in the study. All participants received the treatment according to their group allocation. Twenty out of 21 (95%) participants completed the study and one participant dropped out after the first visit due to a family matter (Fig. 1).

Because of the low recruitment rate of male participants during the first half of the recruitment period, our initial plan of recruiting an equal number of male (10) and female (10) participants was revised to recruit a total of 20 participants regardless of sex. During the study period, an additional randomization list of 20 equally weighted allocations was generated and uploaded to REDCAP in order to have enough group allocations for female participants.

### Protocol deviations

Pressure pain threshold was abandoned prior to the start of the study due to concerns during preparatory pilot testing that this additional testing might influence participants’ pain levels. Therefore this outcome was not assessed or collected for any participants. There were no other changes to the protocol, which was completed as described in the trial registry.

### Missing data

Data imputation was not performed for any missing values. Other than the missing data from the participant who dropped out after 24 h, all data were present and analyzed with the following exceptions: missing PFGS measure for one participant (EA group) due to hard-drive crash, several missing entries for pain rating from pain diaries (9 missing out of 180, randomly distributed among participants and time points), and 2 missing entries on caffeine intake during the follow-up period.

### Participant characteristics

The baseline characteristics of the participants are summarized in Table 1. Random, sex-stratified allocation resulted in two groups with equivalent characteristics for all measured parameters.

**Table 1:** Baseline demographic and clinical characteristics

Baseline	EA group	MA group
Age (years)	46 (9.2)	47 (7.5)
Sex (male/female)	5/5	4/7
Duration (months)	6 (29)^a^	5.2 (3.2)
Location of LE (dominant/non-dominant arm)	6/4	11/0
Employment (employed/not employed)	8/2	7/4
Employment involves repeated wrist/arm movements (yes/no)	5/3	5/2
Heavy lifting & carrying (yes/no)	1/7	2/5
Sports (yes/no)	7/3	8/3
TSK-11 (out of 44)	28 (6.3)	26 (6.1)
PRTEE (total) (out of 100)	52 (21)	50 (20)
PRTEE (subscale: pain)	27 (9.9)	27 (10)
PRTEE (subscale: specific activities)	31 (15)	19 (29)^a^
PRTEE (subscale: usual activities)	20 (9.7)	17 (8.6)
PFGS of unaffected arm (N)	340 (130)	310 (120)
PFGS of affected arm (N)	110 (130)^a^	76 (100)^a^
NRS	4.7 (3.6)	4.1 (1.5)

Values are expressed as mean (standard deviation) except^a^, expressed as median (interquartile range) due to asymmetrical distribution of data. N = 21, 20 and 20 for the three time points shown. Error bars represent 95% CI

*NRS* numeric rating scale

### Adverse effects

The study was completed without any significant adverse or side effects. Six participants (five in EA; one in MA) reported post-treatment soreness at 24 h follow up. Post treatment-soreness gradually disappeared in all participants by their second follow up at 72 h. One participant in the MA group reported a mild bruising in the needling region.

### Non-protocol treatments

All participants answered that they did not take NSAIDS or any other painkillers for pain control nor receive any therapy during the study period. All participants in the EA group and all but one in the MA group reported that they avoided any activities that aggravated their elbow pain.

### Outcome measures

There were no differences in pain (NRS) or pain threshold (PFGS) between the EA and MA groups at any time point, either before or after acupuncture treatment. In the entire group of 20 participants, 11 experienced an immediate reduction in pain, 8 stayed the same, and 1 participant’s pain worsened. There was no apparent trend toward improvements being experienced in one group or the other (EA vs. MA). Of the 11 who experienced a reduction in pain, the reduction was at least 2 points on the NRS for 7 participants.

**Fig. 2:**
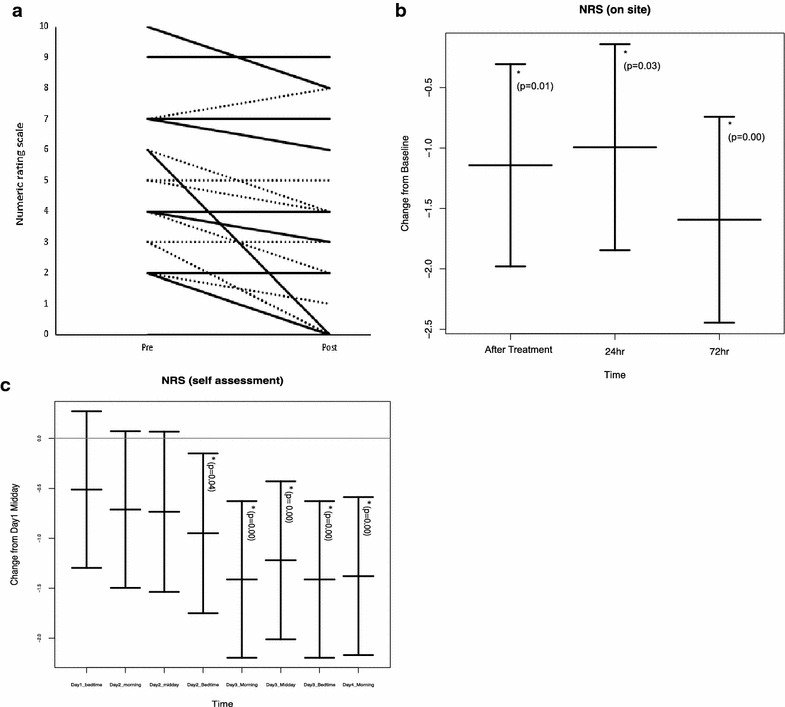
Changes in LE pain over time. **a** Participant-reported pain level before (pre) and after (post) treatment with manual acupuncture (*dotted line*) or electroacupuncture (*solid line*). There was a consistent trend for pain to lessen immediately after treatment, regardless of type of acupuncture. **b** The change in pain from baseline is indicated, with negative numbers indicating a lessening of pain. *Asterisks* indicate statistically significant improvements in pain compared to baseline. *NRS* numeric rating scale. N = 21, 20 and 20 for the three time points shown. *Error bars* represent 95% CI. **c** The pain diary measures demonstrated that, unexpectedly and for unknown reasons, pain levels continued to decline during the 72-h follow-up period. Changes are shown relative to day 1 midday, which was the first diary entry following acupuncture treatment, which took place in the morning of day 1. NRS: Numeric rating scale. N = 20 for all time points shown. *Error bars* represent 95% CI

There was a statistically significant change in the perceived pain level over time, reflected both in onsite measurements (Fig. 2a, b) and in the pain diary (Fig. 2c). Onsite measurements showed that this reduction in pain occurred immediately after treatment (i.e. while the participant was still lying on the treatment plinth), and it appeared to be maintained, on average but with some intra-individual variation, throughout the 72-h follow-up period (Fig. 2c). The diary ratings of pain intensity indicated that from day 1 midday (after treatment), average pain severity continued (unexpectedly) to decrease during the follow-up period.

**Fig. 3:**
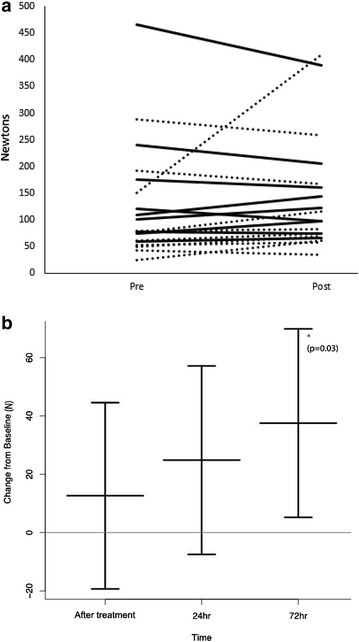
Changes in pain free grip strength (PFGS) over time. **a** The sensory threshold at which point participants report the onset of pain during gripping, before (pre) and after (post) treatment with manual acupuncture (*dotted line*) or electroacupuncture (*solid line*). There was no consistent trend observed for either type of acupuncture, and no difference between the two treatment groups. **b** The change in PFGS from baseline is indicated for all participants (both treatment groups), with positive numbers indicating an improved (raised) pain threshold. Unexpectedly and for unknown reasons, PFGS improved during the 72-h follow-up period. *Asterisk* indicates a statistically significant improvement in PFGS compared to baseline, but this occurred too late in the experiment to be confidently attributed to the effects of acupuncture. N = 21, 20 and 20 for the three time points shown. *Error bars* represent 95% CI

Unlike pain, there was no consistent change in PFGS immediately following treatment (Fig. 3a, b). However, a pattern of a gradual increase in PFGS was observed over the 72-h follow-up period (Fig. 3b), and a significant improvement of PFGS was demonstrated by 72 h follow-up.

With regard to the participants’ global rating of change, 24 h after treatment the majority of participants reported feeling at least ‘slightly better’ (67% in EA and 54% in MA), and one subject (MA) reported feeling much better.

### Efficacy of blinding

Ten out of eleven participants in the MA group answered that they received EA treatment with confidence levels of ‘very confident’ (60%), ‘neutral’ (30%) and ‘somewhat confident’ (10%), indicating that allocation blinding was successful in 91% of the participants. None of these ten participants had previously experienced EA or electro-stimulation. One participant in the MA group who guessed her treatment allocation correctly (with a confidence level of ‘neutral’) had previous experience with electro-stimulation treatments. Thus, the method of sham EA used in the current study was effective as long as the participants did not have previous experience with electroacupuncture or electro-stimulation treatment before. All participants who were allocated to EA guessed correctly that they had received EA with confidence levels of ‘very confident’ (67%) and ‘confident’ (33%).

The assessor guessed the participants’ group allocations correctly 45% of the time (with two ‘somewhat confident’ and seven ‘not confident at all’). Therefore, it can be concluded that the assessor (EB) was successfully blinded to the participants’ group allocation.

## Discussion

This is the first study investigating the simultaneous time course of pain relief and pain-free grip strength in patients with LE after a single acupuncture treatment (MA or EA). It is only the second study, to our knowledge, which has examined the acute time course of LE pain relief following a single acupuncture treatment [36]. Our study was completed without any significant change in methods & procedure, or significant adverse effects. We found that, although there was a small but noticeable improvement in perceived pain immediately following acupuncture treatment, there was not a simultaneous improvement in PFGS. Therefore, acupuncture cannot be recommended as a method to improve pain threshold during gripping for people with LE. Although this is a small study, it nevertheless may have implications for clinical reasoning when prescribing rehabilitation exercises in conjunction with acupuncture.

Our results are consistent with many other trials which have demonstrated that, on average, the acute effects of acupuncture typically result in a small reduction of pain—in our study the mean improvement was 1.2, 95% CI 0.45–1.9, which is very similar to the magnitude of effect observed in recent systematic review including 3025 participants in 13 clinical trials [50]. Mechanistic studies have shown that this effect, at least in a model of experimental pain, is dependent on an expectation of benefit by the recipient, and is of similar magnitude to the placebo response [31]. At an individual level, one third (7 of 21) participants in our study experienced an immediate reduction in pain intensity of at least 30%, which has been proposed as a provisional benchmark for a moderately important clinical change [51].

While our experimental study was not intended to assess the clinical importance of acupuncture in the management of LE, we are intrigued that our results are so divergent from Molsberger et al. [36], a widely cited study which reaches very different conclusions than the current study. In that study, 19 out of 24 who received a *verum* treatment (needling of a non-segmental point, GB34, on the leg for 5 min) obtained significant pain relief (≥50% reduction) immediately after treatment compared to only 6/21 participants in our study who received needling on the same point, GB 34, during the treatment. While there are many differences between our study and that by Molsberger et al. perhaps the most salient is the method of pain assessment: In the trial by Molsberger et al. an examiner “assisted the patient in evaluating his or her personal pain level by physical assessment of the elbow with respect to pressure, load, or movements of the forearm which were causing elbow pain.” In comparison, we simply asked participants to make a fist and rate the level of pain, without any need for assistance by the examiner—this method was successful at provoking pain in 20 of the 21 participants, and the internal consistency of the magnitude of provoked pain can be observed in Fig. 2, as can the consistent occurrence of a small hypoalgesic effect. We suggest that the methods of pain assessment used in the current study are less prone to bias, and may be providing more accurate results than the methods used in the Molsberger et al. trial [36].

In this double-blind, randomized pilot experiment, no significant difference was detected between two types of acupuncture at any time point during a 72-h period, for any outcome measure. This result is similar to a previous study conducted in people with OA of the knee [52] in which the effect of a single treatment of EA vs. MA on pain and strength was examined. As in the current study, both EA and MA provided a similar, significant improvement in pain intensity after a single treatment, but no difference in strength. Similar to our findings, there were no significant differences between EA and MA with regard to pain or strength [52]. It could be speculated that a longer course of treatment may provide different results, as was suggested by Leung and Tsui [53], who investigated the effect of EA and MA on patients with LE after a course of treatments (6 treatments over 2 weeks) and concluded that EA produced significantly greater pain relief and improvement of PFGS compared to MA. However, that study did not report the confidence intervals for the effect size—visual inspection of the published results of that study indicates that a meaningful difference between the EA and MA groups, in terms of change in pain or PFGS, is unlikely. In addition, that study failed to describe the randomization method and did not blind the clinician or the participants. Based on the findings of our study as well as the previous study by Tsui et al. [53], it is unlikely that a single treatment of EA will provide any additional benefit over MA.

Statistically significant improvements in PFGS and perceived pain level were observed in both groups over a 72-h period after one treatment of EA or MA. This was unexpected, because a previous study reported maximum pain relief immediately after needling, followed by a gradual reduction in the therapeutic effect over the ensuring 72 h [36]—the exact opposite of what was observed here. The divergent time course in the two main outcomes (immediate pain relief, followed by more gradual improvement in PFGS) suggests that there may be more than one mechanism underlying these changes. It is likely that, because our participants were instructed to refrain from aggravating activities during the follow-up period, their tendon became gradually less irritable during the follow-up period.

Unlike the pain ratings, significant changes were not observed in the GROC scale. For GROC, ‘much better’ and ‘moderately better’ were considered as success; despite anticipating a small improvement in pain levels, we did not expect that a single needling treatment would result in clinical success. At 24 h follow-up, only one participant reported feeling ‘much better’ and none ‘moderately better’, therefore it can be concluded that a single acupuncture treatment is unlikely to provide substantial improvements for most patients 24 h following treatment. Also, 25% of participants reported feeling worse after the treatment at 24 h follow up for unexplained reasons, perhaps due to the fluctuating nature of LE pain.

A variety of factors can influence individuals’ pain and disability experiences. Although kinesiophobia did not significantly influence the outcomes of this study, previous studies have suggested that anxiety and depression might influence an individual’s pain perception [54, 55]. Considering that patients with LE have shown significantly increased levels of anxiety and depression due to pain and functional disability of the arm [56], the results of this study might have been influenced by participants’ psychosocial status. In addition to controlling for various elements of the placebo response, future studies may wish to examine the impact of anxiety and depression levels on acupuncture-induced hypoalgesia in LE.

There were some limitations in this study: (1) There was no ‘no treatment’ control group in this study. However, given that there was no immediate change in PFGS following needling, we can nevertheless be confident that acupuncture does not have an immediate impact on this outcome, and therefore reject this part of the hypothesis. (2) Over a 72-h period, a pattern of gradual increase in PFGS was seen. Whether PFGS would further increase or decline after a 72-h period is not known as the study ended at 72 h follow up. (3) This study provided one treatment only, and did not include a placebo control (i.e. sham needling). Given that LE is a chronic condition that typically requires several weeks to demonstrate improvement with conservative treatment, the results of this study do not address the relative effectiveness of EA or MA in treatment of LE as it is typically provided (several times a week for several weeks). Nevertheless, the results of this study do suggest that there is no substantial difference between EA and MA with regard to the time course of pain relief and improvement of PFGS after one treatment, which was the hypothesis being tested in this experiment.

## Conclusions

Although acupuncture resulted in noticeable improvements in pain immediately following treatment, this was not accompanied by a simultaneous change in PFGS. The use of acupuncture or electroacupuncture, as administered in the current study, is unlikely to acutely enhance the ability of people with LE to engage in pain-free rehabilitation exercise (Additional file 2: Table S1).

## Additional files

**Additional file 1.** Minimum standards of reporting checklist.

**Additional file 2: Table S1.** Additional table.

## Abbreviations

EA: electro-acupuncture
GROC: global rating of change
LE: lateral epicondylalgia
MA: manual acupuncture (no electricity)
NRS: numeric rating scale
NSAIDs: non-steroidal anti-inflammatory drugs
PFGS: pain-free grip strength
PRP: platelet rich plasma
TSK: Tampa Scale of Kinesiophobia
